# The Physicians’ Visiting List

**Published:** 1913-12

**Authors:** 


### Books and Magazines.

The Physicians' Visiting List.—(Lindsay & Blakiston's, for 1914.) Sixty-third years of its publication. Philadelphia. P. Blakiston's Son & Co., successors to Lindsay & Blakiston, 1012 Walnut Street.

This old favorite contains the special memorandum pages for each month ruled for each day, and for "amount" and ledger page, interleaved for 25 patients a week. It contains also a number of pages for miscellaneous memoranda, for addresses of patients, adresses of nurses, a record of bills and accounts asked for, vaccination engagements, obstetric engagements, a record of births, a record of deaths, ruled also for cash account. In addition to all this there is a calendar, a table for calculating the period of utergestation, a table of signs, incompatibles, poisons, the metric system and table for converting apothecaries' weights and measures into grams, also "the physicians' Dose Table. And the price of all this is $1.25, with a lead pencil thrown in.

